# Hyaluronic Acid Nanoparticles with Parameters Required for *In Vivo* Applications: From Synthesis to Parametrization

**DOI:** 10.1021/acs.biomac.4c00370

**Published:** 2024-06-29

**Authors:** Nikola Matějková, Lucie Korecká, Petr Šálek, Olga Kočková, Ewa Pavlova, Jitka Kašparová, Radka Obořilová, Zdeněk Farka, Karel Frolich, Martin Adam, Anna Carrillo, Zuzana Šinkorová, Zuzana Bílková

**Affiliations:** † Department of Biological and Biochemical Sciences, Faculty of Chemical Technology, University of Pardubice, Studentská 573, Pardubice 532 10, Czech Republic; ‡ Institute of Macromolecular Chemistry, Czech Academy of Sciences, Heyrovského nám. 2, Praha 6 162 00, Czech Republic; § Central European Institute of Technology, Masaryk University, Kamenice 5, Brno 625 00, Czech Republic; ∥ Department of Biochemistry, Faculty of Science, Masaryk University, Kamenice 5, Brno 625 00, Czech Republic; ⊥ Department of Physical Chemistry, Faculty of Chemical Technology, University of Pardubice, Studentská 573, Pardubice 532 10, Czech Republic; # Department of Analytical Chemistry, Faculty of Chemical Technology, University of Pardubice, Studentská 573, Pardubice 532 10, Czech Republic; ∇ Department of Radiobiology, Faculty of Military Health Sciences, University of Defence, Třebešská 1575, Hradec Králové 500 01, Czech Republic

## Abstract

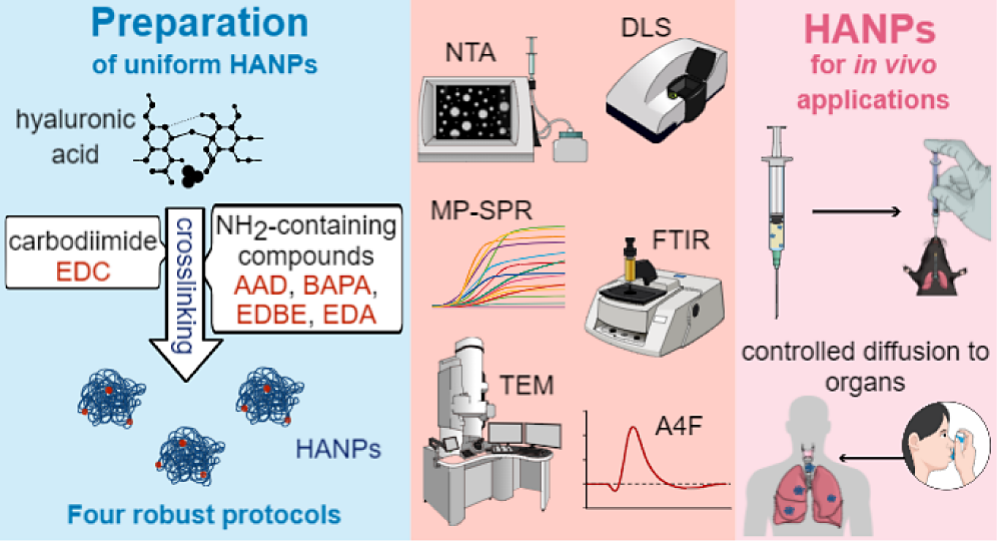

Hyaluronic acid is an excellent biocompatible material for *in vivo* applications. Its ability to bind CD44, a cell receptor involved in numerous biological processes, predetermines HA-based nanomaterials as unique carrier for therapeutic and theranostic applications. Although numerous methods for the synthesis of hyaluronic acid nanoparticles (HANPs) are available today, their low reproducibility and wide size distribution hinder the precise assessment of the effect on the organism. A robust and reproducible approach for producing HANPs that meet strict criteria for *in vivo* applications (*e.g.*, to lung parenchyma) remains challenging. We designed and evaluated four protocols for the preparation of HANPs with those required parameters. The HA molecule was cross-linked by novel combinations of carbodiimide, and four different amine-containing compounds resulted in monodisperse HANPs with a low polydispersity index. By a complex postsynthetic characterization, we confirmed that the prepared HANPs meet the criteria for inhaled therapeutic delivery and other *in vivo* applications.

## Introduction

Recently, advances in material science have enabled us to study and carry out trials of nanocarrier-based imaging and therapeutic systems. They have the potential for use in biomedical applications, such as injury treatment, cancer therapy, tissue regeneration, vision care, drug delivery systems, theranostics, and diagnostics.^1,2^ However, the quality and *in vivo* usability strongly depend on their biocompatibility and controlled biodegradability.^3^ Therefore, nanomaterials based on biopolymers, such as hyaluronic acid (HA),^4^ chitosan,^5,6^ polylactic acid,^7^ poly(lactic-*co*-glycolic acid),^8^ and polycaprolactone,^9^ have attracted the attention of researchers in the field of biomedicine.^10^

HA, a natural linear polysaccharide, comprises repeated disaccharide units of d-glucuronate and *N*-acetyl-d-glucosamine.^11^ The molecular weight of HA present in the human body is between 100 kDa and 8 MDa.^12^ The biological properties of HA depend on its molecular weight; low-molecular-weight HA (4–200 kDa) is an immune stimulant and exhibits angiogenic activity. Medium-molecular-weight HA (200–500 kDa) helps with wound healing and embryonic development. High-molecular-weight HA (>500 kDa) shows high antimicrobial, anti-inflammatory, and antiangiogenic properties.^13^ The encapsulation of high-molecular-weight HA in nanoparticles prevents its natural degradation and offers a nanomaterial with many advantages.

HA can be used as a coating/carrier for cytotoxic drugs, bioactive molecules, and nonbiocompatible nanomaterials.^14−21^ Hyaluronic acid nanoparticles (HANPs) can have therapeutic effects even without carrying the active molecules; they are used to modulate the inflammatory process in the human body.^4^ This effect has been described in the treatment of Type 2 diabetes,^22^ osteoarthritis,^23^ rheumatoid arthritis,^4^ or atherosclerosis.^24^ In our previous work, we reported that HANPs significantly affected molecular and cellular pathways related to radiation-induced pulmonary injuries and radiation-induced processes in lung tissue.^25^ Therefore, the use of HANPs as preradiation therapy in cancer patients could protect lung tissue and prevent the onset of lung fibrosis. However, more data are being collected to fully understand the radioprotective properties of HANPs.

The antimicrobial activity of HANPs in combination with antibiotic delivery is used as efficient therapeutics,^26^ for example, HA–chitosan compounds loaded with antibiotics for the treatment of *Staphylococcus aureus*,^27^ and HANPs loaded with levofloxacin show a significant higher effect on intracellular bacteria than free levofloxacin,^28^ and many other applications.^29^ HANPs can be further decorated with bioactive molecules for targeted delivery or suppressing nanoparticle interception by reticuloendothelial cells.^30^ HANPs can also be used to deliver chemotherapeutic agents to target cancer cells (*e.g.*, colon, gastric, breast, and prostate cancer) where the receptor CD44 is overexpressed.^31−33^ HA naturally binds to CD44, a transmembrane glycoprotein, which plays a crucial role in morphogenesis, apoptosis, tumor metastasis and invasion, and angiogenesis.^30,34^ CD44 is also present on the surface of hematopoietic cells and tissues, such as the central nervous system and the low respiratory tract. Therefore, we can consider HANPs as a unique nanomaterial with therapeutic potential that offers multiple advantages in the case of *in vivo* applications, such as tumor-targeted navigator^35^ and drug/gene delivery systems.^14,36−38^

Targeted transport of HANPs, for example, to the upper or lower respiratory tract, is one of the ways to make full use of the material potential. However, high demands are made on the quality of the particles, their size, dispersity, stability, and controlled biodegradability. These strict criteria are closely related to the anatomy of lung tissue. HANPs must be of suitable aerodynamic sizes to reach the deep lung in the form of an aerosol. Nanoparticles sized up to 100–200 nm penetrate best up to individual alveoli and respiratory mucus.^39^ Only such nanoparticles can overcome the barrier properties of respiratory mucus and penetrate the mucus to approach the underlying airway epithelium. Therefore, it is obvious that the size or dispersion of the particles leads to a limitation of penetration into the microstructures of the lungs and a loss of control over therapeutic processes.^40^ Moreover, particles of >10 μm are trapped in the upper respiratory tract from which they are partially coughed or blown out. Particles with a size of ∼4–10 μm move below the larynx into the lower respiratory tract, and particles of ∼2.5 μm in size easily penetrate the bronchi. Nanoparticles with sizes in the order of tens of nanometers penetrate the alveoli of the lungs, from where they easily enter the bloodstream with the gases. Thus, particles <100 nm are deposited in the alveolar region of the lungs (*i.e.*, alveoli).^40,41^

Various approaches have been used to synthesize HANPs,^42^ including self-assembly,^30^ covalent cross-linking,^36^ and ionic interaction.^37^ The self-assembly process is widely used for the encapsulation of hydrophobic drugs in an HA-based hydrophilic shell. The facile method for the formation of zero-length covalent cross-links uses carbodiimide to activate carboxyl groups on the HA chain and amine to create amide bonds through the linear chains of HA.^42−44^ The formation of nanoparticles through multiple ionic interactions combines polyanionic HA molecules with polycations (*e.g.*, collagen, chitosan, poly-ε-caprolactone).^45,46^

Although various protocols to produce HANPs for *in vivo* applications have been published,^22,23,42 −44,47,48^ their parametrization is often insufficient and unsupported properly by experimental data. Only the combination of various comprehensive techniques can reveal complex information on the parameters of polysaccharide-based nanoassemblies.^49^ The hydrodynamic diameter, the polydispersity index (PDI), and the zeta potential are key parameters providing valuable information on the overall characterization.

Our innovative approach is based on the ability of various amine-containing reactive compounds to form stable covalent bonds with the carboxyl functional groups present in the HA chain. Four amine-containing compounds, adipic acid dihydrazide (AAD; Figure S1), bis(3-aminopropyl)amine (BAPA; Figure S2), 2,2′-(ethylenedioxy)bis(ethylamine) (EDBE; Figure S3), and triethylamine (TEA; Figure S4), subsequently exchanged with ethylenediamine (EDA; Figure S5), were selected. We designed and experimentally validated four protocols to produce HANPs of uniform size and low dispersity suitable for *in vivo* applications, *e.g.*, for inhaled therapeutic delivery.

## Materials and Methods

### Materials

HA sodium salt from *Streptococcus equi* (1.5–1.8 MDa), *N*-(3-(dimethylamino)propyl)-*N*′-ethylcarbodiimide hydrochloride (EDC), adipic acid dihydrazide (AAD), bis(3-aminopropyl)amine (BAPA), 2,2′-(ethylenedioxy)bis(ethylamine) (EDBE), triethylamine (TEA), ethylenediamine (EDA), *N*-hydroxysulfosuccinimide sodium salt (sulfo-NHS), *N*-hydroxysuccinimide (NHS), 1-[3-(dimethylamino)propyl]-3-ethylcarbodiimide methiodide (EDCm), acrylamide, *N*,*N*′-methylenebis(acrylamide), *N*,*N*,*N*′,*N*′-tetramethylethylenediamine, ammonium persulfate, potassium dichromate, nitric acid, bromophenol blue, alcian blue, silver nitrate, formaldehyde, Tween 20, and CD44 (Fc fusion; 48.7 kDa) were purchased from Sigma–Aldrich, USA. Sucrose was obtained from SERVA Electrophoresis, Germany. Acetone, sodium carbonate (anhydrous), and other chemicals were of reagent grade and were supplied by PENTA Chemicals, Czech Republic. Planar carboxymethyl dextran (CMDP) surface plasmon resonance (SPR) sensor chips were purchased from XanTec Bioanalytics, Germany.

### Preparation of HANPs

HANPs were prepared *via* intramolecular covalent cross-linking of the carboxyl groups of the linear HA polymer with amine-containing compounds (AAD, BAPA, EDBE, and EDA; illustrative scheme in Figure S6). EDC (Figure S6), a zero-length cross-linking agent, was selected as the smallest reagent for the formation of amide bonds. The carboxylic anhydride intermediate initiated the condensation of a primary amine with carboxylic acid. Protocol no. 1 utilizing the AAD is based on the already published method^43^ with significant modifications for our purposes.

First, acetone (2.04 mL) was added dropwise to a 1.2-mL HA stock solution [2.5 mg mL^–1^ in deionized distilled water (ddH_2_O)]. The solution was incubated for 15 min at room temperature, rotating at a slow speed of 13 rpm, followed by the addition of cross-linking agents. The EDC solution (1.2 mg/30 μL ddH_2_O) was slowly added dropwise, followed immediately by the addition of the respective amine-containing solution: 1.2 mg AAD/30 μL ddH_2_O for Protocol no. 1 (AAD-HANPs); 5 μL BAPA, 1% (v/v) in ddH_2_O for Protocol no. 2 (BAPA-HANPs); 5 μL EDBE, 1% (v/v) in ddH_2_O for Protocol no. 3 (EDBE-HANPs), and 5 μL EDA, 1% (v/v) in ddH_2_O for Protocol no. 4 (EDA-HANPs). The reaction mixture was incubated for 30 min at room temperature by rotating at a slow speed of 13 rpm. After this incubation, the three-step dropwise addition of acetone (1.22 mL in each step; 30 min incubation in room temperature on slow-speed rotation between steps) was followed. The excess acetone was reduced to a quarter by concentrating under a vacuum (concentrator RCV 2-18 CDplus, Martin Christ Gefriertrocknungsanlagen, Germany) for 15 min at 50 °C. The suspension of the nanoparticles was dialyzed into 0.9% NaCl for 24 h to remove the remaining acetone. The dialysis solution was changed after 1–2 h of dialysis.

### Characterization of HANPs

#### Dynamic Light Scattering

For analysis, 1.5 mL of HANPs suspension (1 mg mL^–1^ in 0.9% NaCl) was transferred to polystyrene cuvettes. Dynamic light scattering (DLS) measurements were performed on the HORIBA SZ-100 instrument (Japan). All samples were measured eight times under the following conditions: 25 °C, 173° detector, and 1.560 as the refractive index for HANPs and 1.333 for 0.9% NaCl solution (dispersion medium). Data were processed with MATLAB software (MathWorks, USA).

#### Nanoparticle Tracking Analysis

Nanoparticle tracking analysis (NTA) was performed using a NanoSight NS300 (Malvern, United Kingdom) instrument containing a sample chamber of ∼1 mL, a 532 nm laser, and a sCMOS camera. The sample was pumped into the chamber with a sterile 1 mL syringe. Each sample was analyzed five times for 15 s with manual adjustment, and all measurements were performed at 25 °C. The analytical software NTA 2.3 Dev Build 3.2.16 was used for data capture and evaluation according to the number-based particle size distribution. Mean sizes (diameters) and standard deviations were used to evaluate the analyzed nanoparticles.

The lyophilized HANPs samples for NTA (2 mg; 1 mg mL^–1^) were dispersed in Milli-Q pure water prepared using a laboratory water purification system Milli-Q IQ 7000 (Merck KGaA, Darmstadt, Germany). The HANPs were dispersed in glass vials using a UP200S ultrasonic processor (Hielscher Ultrasonics GmbH, Germany) for 5 min at 40% amplitude using the described method: 2 min with pulsation (0.5 s pulse rate), 2 min without pulsation, and 1 min with pulsation (0.5 s pulse rate). Each sample was diluted with Milli-Q water at a concentration of 50 μg mL^–1^. Approximately 0.8 mL of the sample was pumped into the chamber with a sterile 1 mL syringe.

#### Zeta Potential Measurement

For the measurement of zeta potential, the HANPs were dialyzed overnight to 0.9% NaCl as stated above or for 1 h to ddH_2_O with three changes of dialysis solution (every 15 min). The zeta potential was measured in the electrode cuvette on the HORIBA SZ-100 instrument (Japan). All samples were measured ten times under the following conditions: run duration of 80 s, accumulated time of 40 s, and delay between measurements 10 s. Data were processed with MATLAB software (MathWorks, USA).

#### Fourier-Transform Infrared Spectroscopy

The samples for Fourier-transform infrared (FTIR) spectroscopy analysis were prepared according to the above-mentioned protocol (preparation of HANPs), followed by complete evaporation using a vacuum concentrator (evaporation for 4–6 h) and measured in the solid phase. IR spectra were recorded using a Nicolet iS50 FT-IR spectrometer equipped with a built-in single-bounce diamond crystal ATR accessory (Thermo Fisher Scientific Co., USA). The dried sample was placed on the ATR crystal and tightened by the pressure arm to fit the sample tightly to the surface of the diamond crystal. All spectra were recorded within the 4000–400 cm^–1^ spectral range with a resolution of 4 cm^–1^. A total of 25 scans were averaged to reduce the noise for each measurement.

#### Asymmetric Flow Field Flow Fractionation

The weight-average molar weights (*M*_w_) and the root-mean-square (RMS) radii of the samples were measured by asymmetric flow field flow fractionation (A4F). The solvent and sample delivery system consisted of an Agilent G1310A pump, a G1322A degasser, and a G1329A autosampler. An A4F long channel (Wyatt, USA), assembled with a 350 μm spacer and a regenerated cellulose membrane with a cutoff of 10 000 g mol^–1^, was used. A Wyatt Optilab-REX RI detector and a Wyatt Dawn 8+ multiangle light-scattering unit were used in series. All system components were controlled by an ECLIPSE 3+ unit (Wyatt, USA).

Milli-Q water was used as a solvent. Sodium azide (NaN_3_; ≥99%, Lach-Ner) was added at a concentration of 0.2 g L^–1^ as an antibacterial agent.

The lyophilized HANPs samples were dissolved and filtered through a 0.45-μm polyvinylidene fluoride (PVDF) filter before injection. Measurements were performed at a constant detector flow rate of 0.6 mL min^–1^. The focusing time was 5 min at a crossflow of 1.5 mL min^–1^ with an injection flow of 0.2 mL min^–1^. The 100-μL sample solution with a concentration of 1 mg mL^–1^ was injected in all cases. After the focusing step, the crossflow was linearly decreased from 1.0 to 0.1 mL min^–1^ in 60 min and maintained at a constant value of 0.1 mL min^–1^ for the next 15 min, followed by 15 min without crossflow. Data were collected and evaluated using the Astra V software, version 5.3.4.15, from Wyatt. A specific refractive index increment (d*n*/d*c*) of 0.15 was used.

#### Transmission Electron Microscopy

Transmission electron microscopy (TEM) micrographs of HANPs were obtained using a TEM Tecnai G2 Spirit Twin 12 (FEI, Czech Republic) using bright field imaging mode at an accelerating voltage of 120 kV.

The lyophilized HANPs were dissolved in Milli-Q pure water to a concentration of 1 mg mL^–1^. 200 μL of the suspension was dropped onto a microscopic copper TEM grid (300 mesh) coated with a thin electron-transparent carbon film. The suspension was allowed to sediment for 10 min, and the excess solution was removed from the bottom of the grid by soaking with filter paper (fast drying method). This fast removal of the solution was performed to minimize oversaturation during drying. The particles were negatively stained with uranyl acetate (2 wt % solution was dropped onto the dried nanoparticles and removed after 15 s as described for the previous solution). The sample was finally completely dried in air at room temperature and observed through the TEM.

The morphology observed by TEM was additionally confirmed by cryogenic TEM (cryo-TEM). The 4 μL sample (lyophilized HANPs diluted using Milli-Q pure water at a concentration of 3 mg mL^–1^) was dropped onto an electron microscopy grid covered with a holey carbon supporting film (Electron Microscopy Science, USA), hydrophilized just before use by glow discharge (Expanded Plasma Cleaner, Harrick Plasma, USA). The excess solution was removed by blotting (Whatman no. 1 filter paper) for 1 s, and the grid was plunged into liquid ethane held at 181–182 °C. The vitrified sample was transferred to the microscope and observed at −173 °C at an accelerating voltage of 120 kV. The analyses of particle size distributions from TEM images were performed using ImageJ software (National Institutes of Health, USA).

#### Multiparametric Surface Plasmon Resonance

The affinity experiments were performed using a multiparametric surface plasmon resonance (MP-SPR) system MP-SPR Navi 200 OTSO (BioNavis, Finland). The experiments were executed using MP-SPR Navi Control software (BioNavis, Finland), with the sensor response evaluated in angular scan mode by a centroid fitting function. The instrument was washed with ddH_2_O, 70% ethanol, and ddH_2_O and degassed 0.9% NaCl (pH 7.4) with 0.05% Tween 20 as a running buffer. The CMDP-modified SPR chip (XanTec bioanalytics GmbH, Germany) was loaded into the instrument, and the baseline signal was established with the running buffer at a flow rate of 20 μL min^–1^. The chip surface was activated using 0.2-M EDC and 0.05-M sulfo-NHS mixture (1:1 v/v). The mixture was injected into each channel for 7 min at a flow rate of 20 μL min^–1^. Amine groups were introduced onto the chip surface by injecting 0.2-M AAD for 5 min at a flow rate of 20 μL min^–1^ to both channels. The receptor CD44 was bound onto the surface of the chip by mixing CD44 with EDC in a mass ratio of 1:10 and diluted to a final concentration of 14 μg CD44 per 700 μL of 10 mM sodium acetate buffer (pH 4.5). The mixture was flown through the sample channel for 40 min at a flow rate of 10 μL min^–1^; only the acetate buffer was injected into the reference channel. All of these reagents were diluted in ddH_2_O. Subsequently, AAD-, BAPA-, EDBE-, and EDA-HANPs and the solution of free HA (1.5–1.8 MDa) diluted with 0.9% NaCl at concentrations of 0.05 mg mL^–1^ and 0.1 mg mL^–1^ (and 0.25 mg mL^–1^ for free HA) were injected into both channels for 10 min at the flow rate of 20 μL min^–1^ with a dissociation time of additional 10 min. The sensor surface was regenerated using 10 mM HCl for 2 min (20 μL min^–1^) between samples. The measured data were evaluated using an MP-SPR Navi DataViewer (BioNavis, Finland).

## Results and Discussion

The main goal of this work was to prepare cross-linked HANPs with variable sizes and low polydispersity. As stated, especially for our intended future use, the size and dispersity of HANPs influence their penetration and deposition on the mucous of the lower respiratory tract. Protocol no. 1 with EDC and AAD cross-linkers to form AAD-HANPs is partially taken from a 2009 US patent.^43^ AAD-HANPs were tested in our previous study for their therapeutic effect during the healing process of the lung mucosa damaged by radiation or chronic inflammation.^25^ We investigated additional cross-linkers: BAPA (Protocol no. 2),^50^ EDBE (Protocol no. 3), and EDA (Protocol no. 4) to monitor the effect of different amine-containing compounds on HANPs.^42,44^ These combinations of amine-containing compounds and EDC were first used for HANPs’ preparation. Therefore, we designed and experimentally validated four protocols of nanoparticle synthesis.

HANPs prepared by following these above-cited protocols were hundreds of nanometers in average size with deviations of ∼14–40%. Thus, we optimized the reaction conditions to prepare HANPs of the desired size, dispersity, high reproducibility, and precision. We modified our protocols by shifting the cross-linking process from an aqueous environment to an organic solvent, in particular acetone, which substantially increased the HANPs’ size uniformity. FTIR analysis (Figure S7) indicates that the cross-linker EDCm does not initiate the quantitative formation of amide bonds, which are characterized by peaks in spectral region of 1560 and 1325 cm^–1^. Exchange for EDC positively affected the number of amide bonds formed. Therefore, EDC as a carbodiimide was used in the four protocols. Insufficient cross-linking is confirmed in HANPs prepared through the cross-linking of EDC with TEA and NHS (Figure S8). The sterically hindered amine group of TEA (Figure S4) limits its participation in the amide bond formation. Thus, TEA was replaced with EDA, which has two reactive side chain amine groups (Figure S5). The analysis indicates that the presence of succinimide (NHS or sulfo-NHS) does not affect the nanoparticle parameters. Therefore, succinimide was not used, considering the biomedical usage of HANPs.

### Dynamic Light Scattering

**Figure 1:**
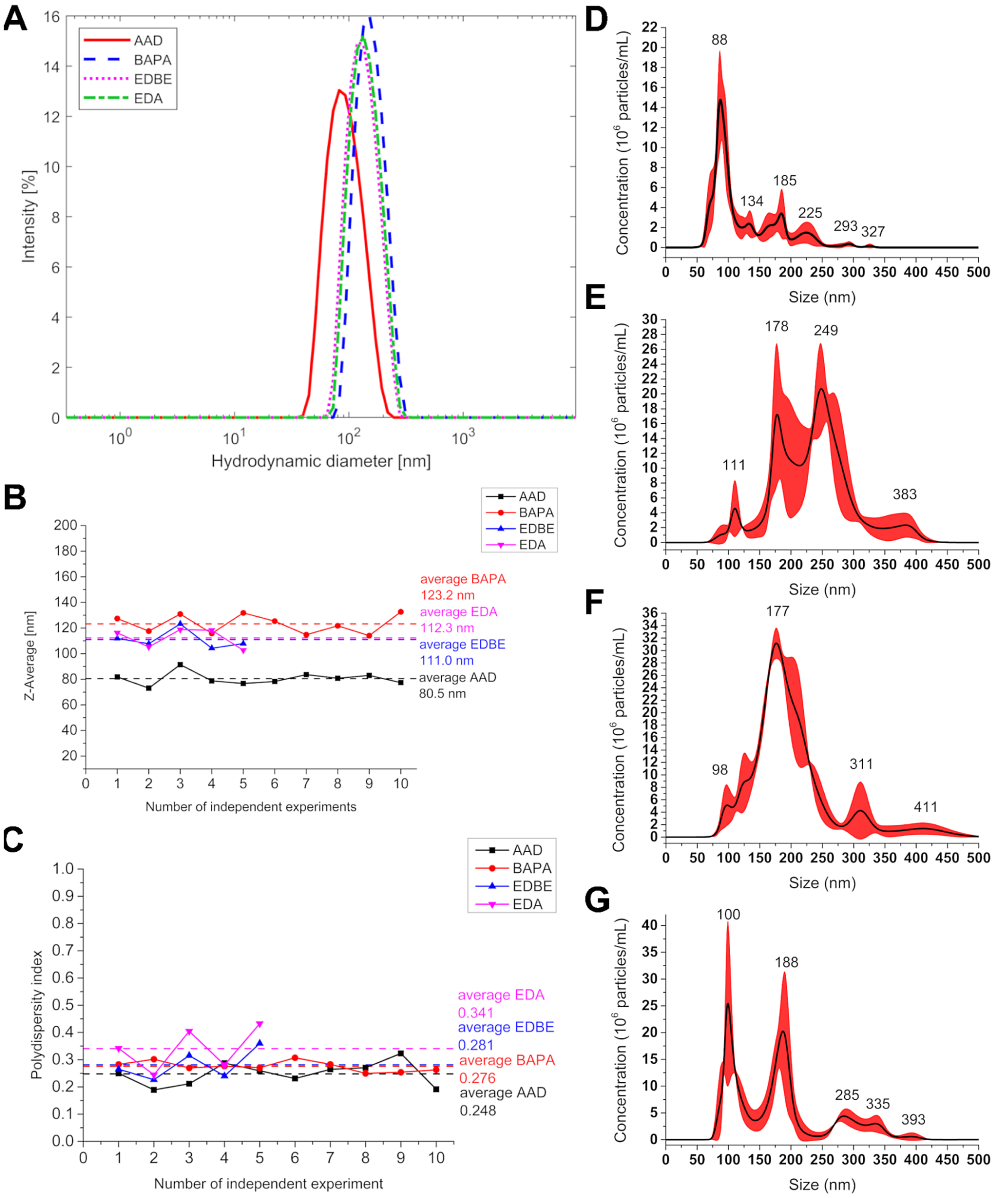
Size of nanoparticles. Average size distribution of HANPs *via* DLS. (A) *z*-average size distribution of AAD-, BAPA-, EDBE-, and EDA-HANPs. Variability of (B) *z*-average and (C) PDI of HANPs with average values (dashed line) between independent synthesis (*n* = 10 for Protocol no. 1 and 2, and *n* = 5 for Protocol no. 3 and 4). NTA average size distribution of HANPs. The red area around the average curve corresponds to the standard deviation between each measurement. (D) Protocol no. 1 (AAD-HANPs), (E) Protocol no. 2 (BAPA-HANPs), (F) Protocol no. 3 (EDBE-HANPs), and (G) Protocol no. 4 (EDA-HANPs).

The cross-linking efficiency and final size of the particles were monitored *via* DLS. AAD-HANPs appear in all repetitions as the smallest with a size (*z*-average: the mean hydrodynamic diameter weighted by intensity) of ∼82 nm. Alternately, BAPA-HANPs are the largest nanoparticles with a size of ∼134 nm. EDBE- and EDA-HANPs are similar, with diameters of ∼112 and 116 nm, respectively (Table 1). The average size distributions of the HANPs are demonstrated in Figure 1A. The size distributions for each protocol are presented in Figure S9.

**Table 1:** Averaged Values of *z*-Average, Mean Hydrodynamic Diameter, and PDI Obtained by DLS, and Mean Hydrodynamic Diameter and Mode Obtained by NTA and Zeta Potential Values of HANPs in 0.9% NaCl and in ddH_2_O^a^

method	variable	AAD-HANPs	BAPA-HANPs	EDBE-HANPs	EDA-HANPs
DLS	*z*-average (nm)	81.8 ± 1.4	134.0 ± 2.0	112.0 ± 0.8	116.3 ± 2.6
	mean hydrodynamic diameter (nm)	90.4 ± 4.3	148.5 ± 3.9	128.9 ± 2.1	135.0 ± 2.8
	polydispersity index	0.25 ± 0.04	0.28 ± 0.04	0.27 ± 0.05	0.34 ± 0.04
NTA	mean hydrodynamic diameter (nm)	124.1 ± 5.2	235.8 ± 9.1	205.1 ± 1.8	190.8 ± 9.8
	mode hydrodynamic diameter (nm)	88.6 ± 2.8	226.3 ± 18.1	185.5 ± 9.1	126.7 ± 25.8
zeta potential	in 0.9% NaCl (mV)	–3.0 ± 0.8	–2.7 ± 0.7	–3.7 ± 1.2	–1.3 ± 0.6
	in ddH_2_O (mV)	–67.3 ± 2.6	–71.4 ± 1.7	–67.3 ± 2.2	–71.2 ± 4.2

a The DLS values with standard deviation were obtained using eight independent syntheses. NTA values of the HANPs obtained following the four different protocols were measured in triplicate. The zeta potential values with standard deviation were obtained using three independent syntheses.

Analytical parameters (repeatability and robustness) obtained through the four protocols were also tested and verified (Figure 1B,C). We repeatedly prepared HANPs (ten, respectively five independent HANPs synthesis for Protocol no. 1 and 2, respectively for no. 3 and 4 in the period of six, respectively three months) and comprehensively characterized them. The *z*-average of (i) AAD-HANPs is between 73.1 and 91.5 nm with a PDI of 0.19–0.29, (ii) BAPA-HANPs is between 114.1 and 132.7 nm with a PDI of 0.25–0.31, (iii) EDBE-HANPs is between 104.2 and 123.2 nm with a PDI of 0.23–0.36, and (iv) EDA-HANPs is between 102.7 and 118.9 nm with a PDI of 0.24–0.43.

DLS is user-friendly, rapid, cheap, and solvent compatible, and was popular in the early years of nanotechnology research. However, this method has shown some limitations throughout the years, such as poor resolution or the tendency to overestimate larger sizes due to the high scattering intensity^51^ and the challenges of polydisperse samples.^52^ Thus, to precisely characterize nanoparticles, other advanced instruments and methods must be implemented.

### Nanoparticle Tracking Analysis

NTA is used to provide accurate and reliable data on the size and uniformity of HANPs. The mean hydrodynamic diameter values and the hydrodynamic diameter mode obtained *via* NTA are listed in Table 1. Even with low polydispersity, the data of the HANPs obtained following each method (DLS and NTA) show different mean hydrodynamic diameters based on different calculations using each method.^53^ The hydrodynamic diameter mode measured by NTA corresponds to the mean hydrodynamic diameter of AAD- and EDA-HANPs obtained *via* DLS. BAPA-HANPs analyzed *via* NTA appear larger than when analyzed *via* DLS. The difference between the average diameters by DLS and NTA could be due to the use of a different dispersion medium for these two measurements, where the BAPA-HANPs were measured in Milli-Q pure water during NTA and might be more swollen compared to the DLS measurements in a 0.9% NaCl solution. This pronounced swelling difference was only observed with BAPA-HANPs prepared according to Protocol no. 2, suggesting that using a BAPA cross-linker compared to three other cross-linkers probably leads to less cross-linked and less compact HANPs, which was confirmed by FTIR. Additionally, NTA revealed two significant populations of HANPs (Figure 1E), which were hidden in a single peak after DLS analysis (Figure 1A). However, both characterization methods indicate that BAPA-HANPs synthesized following Protocol no. 2 are the largest nanoparticles among the four types. EDBE-HANPs show larger sizes *via* NTA than those analyzed *via* DLS, which could be caused by the formation of a small fraction of aggregates (Figure 1F). Protocol no. 4 also indicates the multimodality of EDA-HANPs (Figure 1G), and NTA also revealed the presence of two significant populations of HANPs as observed with BAPA-HANPs prepared by Protocol no. 2.

### Zeta Potential

To evaluate the surface charge of HANPs, the zeta potential measurements were performed. The potential was first measured in 0.9% NaCl, in which HANPs are stable and suitable for *in vitro*/*in vivo* administration. However, as the data in Table 1 show, the strong negative charge of HA is mostly compensated for by salt ions. Therefore, the zeta potential of HANPs was also measured in ddH_2_O to closely evaluate the true surface charge of the nanoparticles. As the results suggest, HANPs themselves have a significant negative charge caused by strongly anionic HA, which is in agreement with the literature.^54,55^

### Fourier-Transform Infrared Spectroscopy

**Figure 2:**
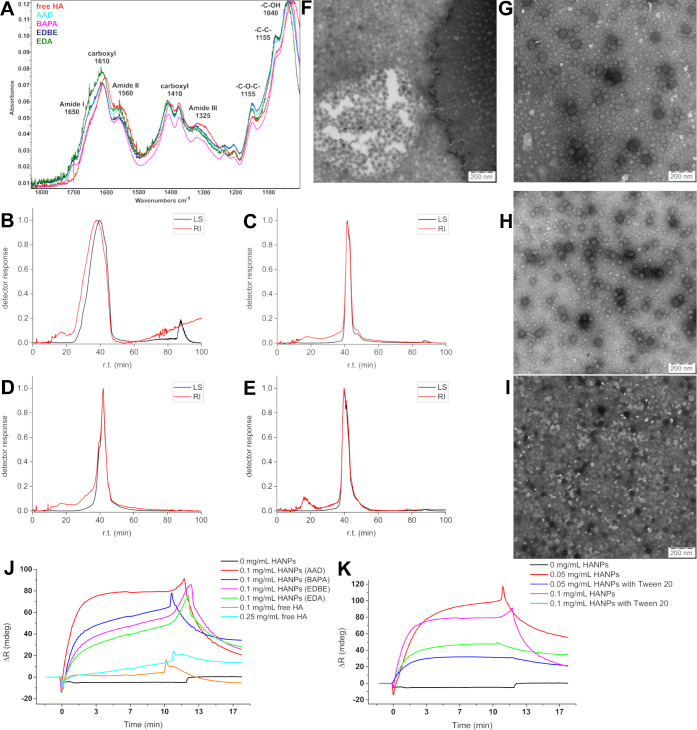
Characteristics of nanoparticles. (A) FTIR spectra in the range between 1830 and 1000 cm^–1^. Comparison of free HA (red spectrum) and AAD-, BAPA-, EDBE-, and EDA-HANPs. Fractograms obtained by A4F of (B) AAD-HANPs, (C) BAPA-HANPs, (D) EDBE-HANPs, and (E) EDA-HANPs. The TEM images of (F) AAD-HANPs, (G) BAPA-HANPs, (H) EDBE-HANPs, and (I) EDA-HANPs stained with uranyl acetate. (J) SPR binding curves of HANPs and free HA on the CD44-modified CMDP SPR chip. (K) SPR binding curves of AAD-HANPs on the CD44-modified CMDP SPR chip demonstrating the effect of 0.05% Tween 20.

The formation of amide bonds among the HA chains was validated *via* FTIR analysis. The cross-linking efficiency of amine-containing compounds was evaluated. The bands of the spectra are assigned by comparing them with the referenced literature (Figure 2A).^56−58^ The spectrum was cropped for the detail of the amide vibrations within the range of 1830–1000 cm^–1^. The complete spectra are presented in Figure S10. The AAD- and EDBE-HANPs are similar based on this characterization. The similarity between the curves of free HA and BAPA-HANPs indicates that these nanoparticles have the smallest degree of cross-linking. BAPA-HANPs have the strongest peak corresponding to the C–OH bond (1040 cm^–1^) and the weakest peak for amides (1560 and 1325 cm^–1^). EDA-HANPs show a shift in the peak positions and the strongest absorbance by the amide peaks, suggesting the highest degree of cross-linking among the HANPs obtained following the four protocols. However, FTIR was not capable of detailed characterization because of the chemical similarity of the samples. The spectra of HANPs prepared following each protocol for nonoptimized and optimized HANPs are presented in Figures S7, S8, S11, and S12.

### Asymmetric Flow Field Flow Fractionation

A4F analysis was performed to study the molecular weight distribution and the averages and molecular dimensions of the HANPs prepared following the four protocols. The obtained mass average molecular weight and diameter (calculated from the root-mean-square radius) of each HANPs fraction are given in Table 2. The HANPs fractograms (Figure 2B–D) contain the main peak comprising two or more unresolved peaks and a minor peak at a lower retention time corresponding to ∼1% by mass. The small initial peak of AAD-HANPs is probably caused by insufficient dialysis. The minor peaks at a lower retention time of the EDBE- and EDA-HANPs probably correspond to free HA or unimolecular nanoparticles (Figure 2C,D). This is also supported by the result of TBE-PAGE (according to the methodology already published^59^), where an increased amount of free HA (*M*_w_ 1.5–1.8 MDa) is evident in the case of particles EDBE- and EDA-HANPs compared to AAD- and BAPA-HANPs (Figure S13). The TBE-PAGE also confirmed no fractionation of HA polymer in the four protocols (Figure S13). The molar mass of the main peak is several times higher, suggesting either the existence of intermolecular cross-linked nanoparticles or the aggregation of unimolecular nanoparticles, which might be promoted by the initial focusing phase of the measurement method. Both these processes agree with the apparent multimodality of the main peaks. The main peak of AAD-HANPs is significantly wider than those of HANPs obtained following the other protocols. This finding indicates some disadvantages of Protocol no. 1 compared to the other methods. Thus, the cross-linking agent should be selected adequately based on the purpose for which HANPs are prepared.

**Table 2:** A4F Measurements: Average Molecular Weights (*M*_w_) and Diameters of HANPs

	AAD-HANPs	BAPA-HANPs	EDBE-HANPs	EDA-HANPs
*M*_w_ (MDa)	5.1	18	11.5	9.6
diameter (nm)	66.0 ± 2.4	96.6 ± 3.3	72.6 ± 2.3	89.4 ± 3.0

### Transmission Electron Microscopy

The images analyzed *via* TEM show spherical HANPs prepared by different cross-linking agents. The average diameters of AAD-HANPs (Figure 2F), BAPA-HANPs (Figure 2G), EDBE-HANPs (Figure 2H), and EDA-HANPs (Figure 2I) are 42.8 ± 6.4, 59.6 ± 5.8, 59.0 ± 7.6, and 40.6 ± 10.1 nm, respectively. The predictable shrinkage of HANPs’ sizes is related to the sample preparation process. For this reason, cryo-TEM measurements of HANPs in the hydrated state were also performed. However, even here, we recorded smaller sizes of HANPs than in other characterization methods, suggesting that neither of the TEM techniques could detect the hydration shell of HANPs, which, in the case of a strong hydrophilic HA biopolymer, is an integral part of the HANPs. Cryo-TEM images are available in Figure S14.

### Multiparametric Surface Plasmon Resonance

MP-SPR is a suitable method to check the biospecific reactivity, in the case of HA, to prove the affinity of cross-linked HANPs to the CD44 receptor for the application of HANPs in drug delivery systems or therapeutics. The CD44 molecules were covalently bound to a carboxymethyl dextran-modified SPR chip to monitor the reactivity between HANPs and the target receptor. The final SPR curves confirm the binding ability of cross-linked HANPs to CD44 (Figure 2J). The curves demonstrate the difference in signal between the specific (with CD44) and nonspecific (the reference channel without CD44) channels. The sensorgram for the entire experiment is shown in Figure S15, studying the interactions of HANPs and free HA. The curves indicate a lower binding affinity of free HA (even at higher concentrations) than those of HANPs. This is due to the larger size of HANPs compared to free HA, resulting in a greater difference in the refractive index per single-bound entity, which could apparently lead to higher signals. However, despite the small size (82 nm *via* DLS), AAD-HANPs show the strongest signal compared to HANPs obtained through the other protocols, suggesting the highest affinity among the studied HANPs. The same experiment was carried out at concentrations of HANPs and HA of 0.05 mg mL^–1^ with similar results (data not shown). The SPR analysis confirmed the potential of HANPs in drug delivery by maintaining their ability to bind to CD44 in the form of nanoparticles.

The addition of Tween 20 to the sample buffer reduced the effect of nonspecific sorption observed during the previous measurements (Figure 2K). Thus, in the presence of Tween 20, the initial binding to CD44 is lower; however, the dissociation of the complex of HANPs with the CD44 receptor is also lower. Therefore, we assumed that the presence of Tween 20 could affect the weak interactions between CD44 and HANPs.

### Stability of HANPs

The HANPs are stable in a 0.9% NaCl solution at 4 °C for several months. However, the composition of the storage liquid, its purity, and sterility are significant for stability. The tendency of HANPs to aggregate is inversely correlated with the molarity of the buffer used to store HANPs. Therefore, lyophilization is an effective tool to stabilize HANPs for long-term storage. The quality of lyophilized and reswelled HANPs in 0.9% NaCl without stabilizing additives was not affected within six independent experiments. There are no fundamental changes in the parameters of the HANPs measured directly after preparation, and the same nanoparticles, which were measured after the subsequent lyophilization and repeated reswelling in 0.9% NaCl. Only a slight change in the diameter measured by DLS was observed (Table 3).

**Table 3:** Differences Between Sizes (*z*-Average) and PDI of Freshly Prepared HANPs and the Same HANPs after Lyophilization and Reswelling

	AAD-HANPs	BAPA-HANPs	EDBE-HANPs	EDA-HANPs
Δ*z*-average (%)	12.11	7.06	6.54	6.69
ΔPDI (%)	6.25	3.13	3.03	8.70

The parameters of HANPs prepared following our four protocols were compared with already published HA-based nanoparticles (Table 4). We prepared uniform spherical nanoparticles with narrow size distribution and low PDI using all four protocols. AAD-HANPs are the smallest nanoparticles, as confirmed by DLS, NTA, and A4F. These HANPs also showed the strongest signal during MP-SPR analysis, suggesting the highest affinity to CD44. For evaluation of the potential of each HANPs as drug delivery carriers, an initial experiment was carried out to estimate the binding capacity of the HANPs. The experiment consists of binding the model molecule (ovalbumin) to the surface of HANPs. This experiment was evaluated by SDS-PAGE (Figure S16), which for AAD-HANPs suggests the lowest binding capacity (70%, which corresponds to 42 μg of ovalbumin on 1 mg of AAD-HANPs). This is in agreement with the literature, where HANPs were loaded with myoglobin with an encapsulation efficiency of 53–92%.^60^ The amount of ovalbumin loaded on AAD-HANPs corresponds to 4.2 wt %. Compared to already published works, for example, for cancer therapy, the reported loading capacity of HA-based nanoparticles was 6.6% for metformin and 4.57% for doxorubicin,^61^ 2.97% for paclitaxel,^62^ and 5.6% for erlotinib.^32 ,63^ Other studies suggested a loading capacity for doxorubicin 14–25.8 wt %,^64^ 9.36% or 12.01%.^65,66^ For chondrocyte targeting, HA-based nanoparticles showed a loading capacity of 4.1% for brucine.^67^ The binding of the Connexin43 mimetic peptide to HA-based nanoparticles, used for the treatment of several retinal ischemic and inflammatory disorders, showed the capacity for adsorption of the peptide 5.35% and for incorporation 3.78%.^68^ These data suggest the assumption that our AAD-HANPs meet the minimum binding capacity criteria and could be used as therapeutics or as radioprotective agents for which they have already been tested.^25^ BAPA-HANPs are the largest nanoparticles, as confirmed by DLS, NTA, and A4F. The NTA size distribution and the TEM images suggest the multimodality of these HANPs. The FTIR peaks of these HANPs exhibit the highest degree of similarity with the spectra of the initial HA, indicating a low degree of cross-linking, consistent with the larger size of HANPs obtained by NTA. However, these nanoparticles exhibit the lowest signal of complex HANPs-CD44 (MP-SPR) and a binding capacity of 78% (4.68 wt %; Figure S16). These parameters suggest that these nanoparticles could be used as carriers for small molecules, which could be incorporated inside the nanoparticles. EDBE-HANPs exhibit similar size (*via* DLS and NTA) and ability to bind to CD44 (MP-SPR) to EDA-HANPs. Furthermore, A4F reveals a small fraction of free HA in the sample, corroborating with the results of TBE-PAGE. The binding capacity of EDBE-HANPs is ∼80% (4.8 wt %), suggesting that these nanoparticles are suitable as therapeutic agents where a size greater than 100 nm is necessary. In addition, these nanoparticles can be modified by active molecules, preferably on the surface. EDA-HANPs exhibit multimodality and a small fraction of free HA by A4F, which was also confirmed by TBE-PAGE. However, the peaks of the FTIR spectrum of EDA-HANPs show distinct strong peaks of amides, indicating a high degree of cross-linking, and the highest binding capacity of 91% (5.46 wt %) has been demonstrated. These characteristics of EDA-HANPs point to their suitability as drug delivery carriers (high binding capacity on the surface of nanoparticles) or therapeutic agents, as the highest degree of cross-linking would prolong the nanoparticles half-life in the organism.

**Table 4:** Summary of HANPs Prepared by Already Published Protocols Compared to Our Established Protocols

nanoparticles	preparation method	mean hydrodynamic diameter (nm)	PDI	number of synthesis/number of measurements	reference
HANPs	self-assembly	221 ± 1		N/A	(23)
HANPs (CDI and EDBE)	cross-linking ratios	two peaks:			(42)
	25%	80 ± 20		(N/A)/3	
		500 ± 70			
	50%	75 ± 30		(N/A)/3	
		420 ± 80			
	100%	110 ± 30		(N/A)/3	
		580 ± 80			
HANPs (EDC and AAD)	cross-linking		sigma value		(43)
	2 h	68		N/A	
	9.5 h	112		N/A	
	20 h	394	0.29	N/A	
HANPs	self-assembly	221.0 ± 3.1	0.104	N/A	(22)
HA-Lys-LA NPs (EDC, TEA) l-lysine methyl ester, LA: lipoic acid	cross-linking substitution of amino groups with LA				(44)
	5%	219	0.27	N/A	
	10%	175	0.12	N/A	
	28%	152	0.16	N/A	
HANPs	self-assembly and cross-linking	389 ± 8	0.074	N/A	(47)
HANPs	self-assembly	293.5	0.108	repeated exp. (no additional information)	(48)
HANPs (AAD)	cross-linking	90.4 ± 4.3	0.25 ± 0.04	10/8	presented work (Protocol no. 1)
HANPs (BAPA)	cross-linking	148.5 ± 3.9	0.28 ± 0.04	10/8	presented work (Protocol no. 2)
HANPs (EDBE)	cross-linking	128.9 ± 2.1	0.27 ± 0.05	5/8	presented work (Protocol no. 3)
HANPs (EDA)	cross-linking	135.0 ± 2.8	0.34 ± 0.04	5/8	presented work (Protocol no. 4)

## Conclusions

This study has presented an improved approach for preparing HANPs with desired parameters for targeted transport to the lower respiratory tract and for other *in vivo* applications, including drug delivery. Novel combinations of cross-linking agents and specific reaction conditions were implemented to prepare HANPs of sizes ranging from 80 to 135 nm with a significantly low polydispersity. The variable size and structure compactness of HANPs affect the appropriateness of their target use, from the ability to pass through mucous barriers and reach the airway epithelium of deep lung tissue to suitability for drug delivery after modification with therapeutic agents. HANPs characterized *via* DLS, NTA, FTIR, A4F, and TEM indicated the formation of uniform nanoparticles with defined sizes and low polydispersity indices. The robustness of the methodology and the reliability of the final product parameters highlight the significance of our study. MP-SPR confirmed that the HANPs maintained the affinity for the CD44 receptor. We believe that HANPs are a proper tool for better understanding the role of CD44 and other key molecules involved in lung parenchyma remodeling and fibrosis, as well as for drug discovery that affects remodeling and fibrosis in multiple types of tissues.^25^

## Supporting Information Available

Chemical structure of AAD (Figure S1); chemical structure of BAPA (Figure S2); chemical structure of EDBE (Figure S3); chemical structure of TEA (Figure S4); chemical structure of EDA (Figure S5); illustrative scheme of cross-linking of HA with EDC and AAD (Figure S6); FTIR spectra of EDBE-HANPs and EDA-HANPs before and after optimizations (Figures S7 and S8); DLS size distribution of HANPs (Figure S9); uncropped original FTIR spectra (Figure S10); FTIR spectra of AAD-HANPs and BAPA-HANPs before and after optimizations (Figures S11 and S12); image of polyacrylamide gel from TBE-PAGE (Figure S13); cryo-TEM images of HANPs (Figure S14); sensorgram of SPR measurement with CD44 (Figure S15); images of polyacrylamide gel from SDS-PAGE (Figure S16) (PDF)

## Abbreviations

A4F: asymmetric flow field flow fractionation
AAD: adipic acid dihydrazide
ATR: attenuated total reflectance
BAPA: bis(3-aminopropyl)amine
CD44: cluster of differentiation 44
CMDP: planar carboxymethyl dextran
cryo-TEM: cryogenic transmission electron microscopy
ddH_2_O: deionized distilled water
DLS: dynamic light scattering
RI: refractive index detector
EDA: ethylenediamine
EDBE: 2,2′-(ethylenedioxy)bis(ethylamine)
EDC: *N*-(3-dimethylaminopropyl)-*N*′-ethylcarbodiimide hydrochloride
EDCm: 1-[3-(dimethylamino)propyl]-3-ethylcarbodiimide methiodide
EDTA: ethylenediamine tetraacetic acid
FTIR: Fourier-transform infrared spectroscopy
HA: hyaluronic acid
HANPs: hyaluronic acid nanoparticles
IgG: immunoglobulin G
MALS: multiangle light-scattering detector
MP-SPR: multiparametric surface plasmon resonance
NHS: *N*-hydroxysuccinimide
NTA: nanoparticle tracking analysis
PDI: polydispersity index
PVDF: polyvinylidene fluoride
RMS: root mean square
SPR: surface plasmon resonance
sulfo-NHS: *N*-hydroxysulfosuccinimide sodium salt
TBE: Tris–borate–EDTA
TBE-PAGE: polyacrylamide gel electrophoresis in Tris–borate–EDTA environment
TEA: triethylamine
TEM: transmission electron microscopy
Tris: tris(hydroxymethyl)aminomethane
UV: ultraviolet
