# Supplementary material for: Hyaluronic Acid Nanoparticles with Parameters Required for In Vivo Applications: From Synthesis to Parametrization
Source: Biomacromolecules. 2024 Jun 29;25(8):4934–45. doi: 10.1021/acs.biomac.4c00370 (PMC11323013; doi:10.1021/acs.biomac.4c00370)
Supplement: Supplementary file 1 — bm4c00370_si_001.pdf [file bm4c00370_si_001.pdf]

Supporting Information

Hyaluronic acid nanoparticles with parameters required for *in vivo* applications: from synthesis to parametrization

*Nikola Matějková[1], Lucie Korecká[*1], Petr Šálek[2], Olga Kočková[2], Ewa Pavlova[2], Jitka Kašparová[1], Radka Obořilová[3,4], Zdeněk Farka[3,4], Karel Frolich[5], Martin Adam[6], Anna Carrillo[7], Zuzana Šinkorová[7] and Zuzana Bílková[1]*

[1]Department of Biological and Biochemical Sciences, Faculty of Chemical Technology, University of Pardubice, Studentská 573, 532 10 Pardubice, Czech Republic

[2]Institute of Macromolecular Chemistry, Czech Academy of Sciences, Heyrovského nám. 2, 162 00 Praha 6, Czech Republic

[3]Central European Institute of Technology, Masaryk University, Kamenice 5, 625 00 Brno, Czech Republic

[4]Department of Biochemistry, Faculty of Science, Masaryk University, Kamenice 5, 625 00 Brno, Czech Republic

[5]Department of Physical Chemistry, Faculty of Chemical Technology, University of Pardubice, Studentská 573, 532 10 Pardubice, Czech Republic

[6]Department of Analytical Chemistry, Faculty of Chemical Technology, University of Pardubice, Studentská 573, 532 10 Pardubice, Czech Republic

[7]Department of Radiobiology, Faculty of Military Health Sciences, University of Defence, Třebešská 1575, 500 01 Hradec Králové, Czech Republic

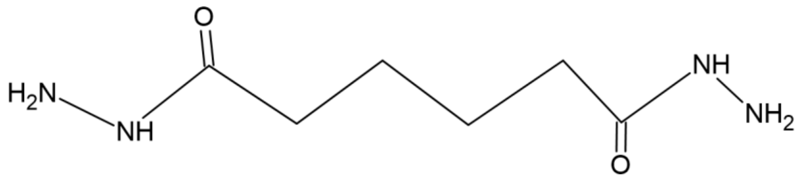

Figure S1. Chemical structure of adipic acid dihydrazide (AAD).

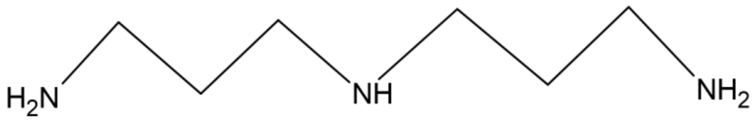

Figure S2. Chemical structure of bis(3-aminopropyl)amine (BAPA).

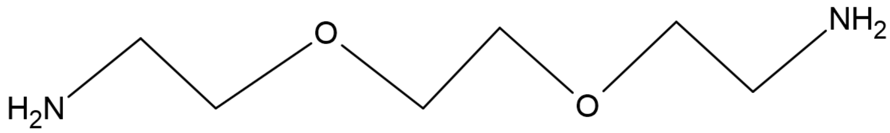

Figure S3. Chemical structure of 2,2′-(ethylenedioxy)bis(ethylamine) (EDBE).

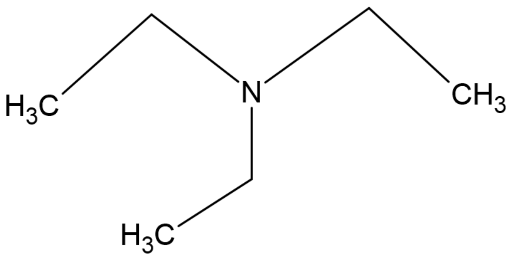

Figure S4. Chemical structure of triethylamine (TEA).

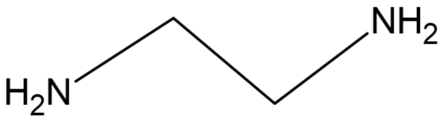

Figure S5. Chemical structure of ethylenediamine (EDA).

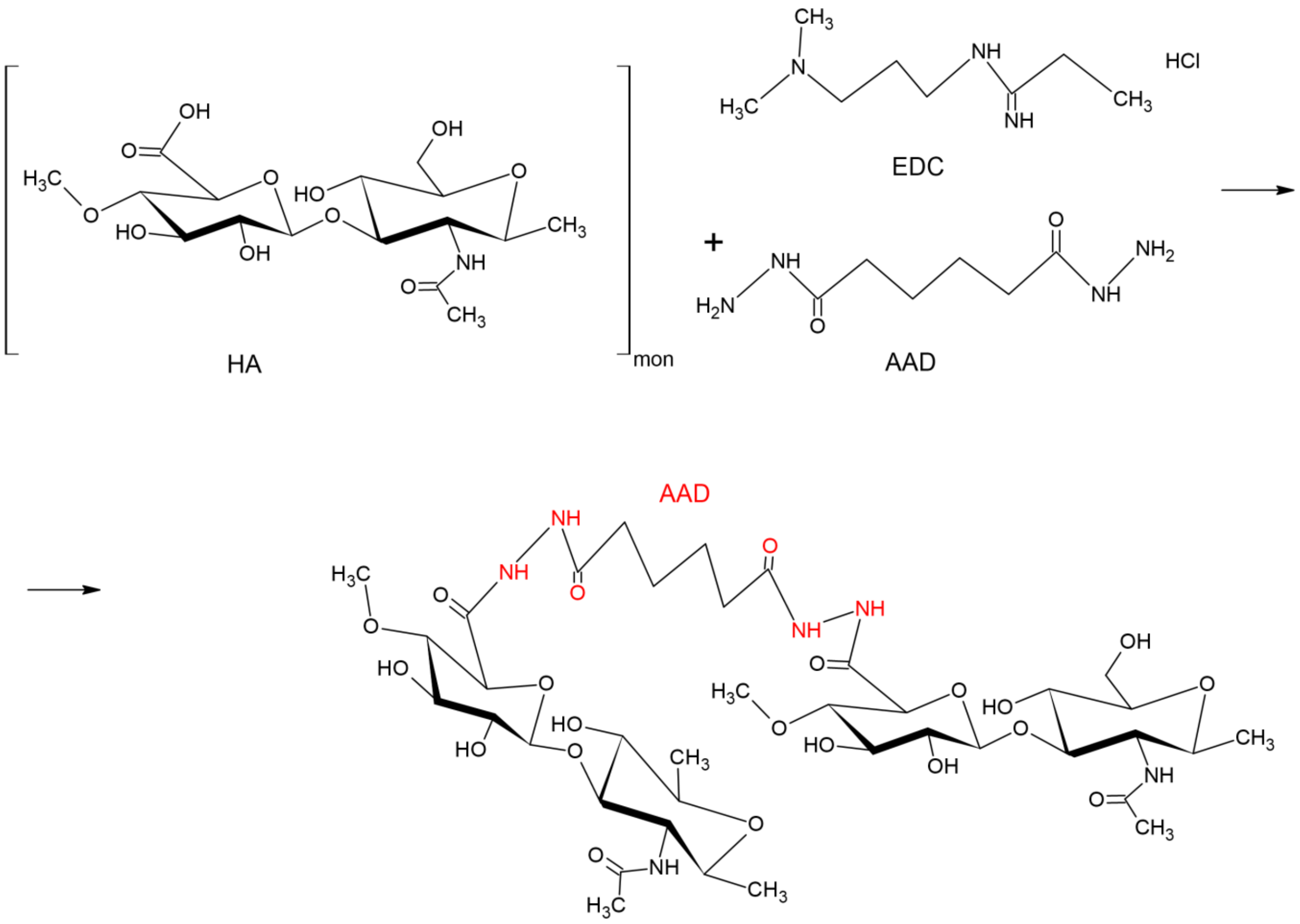

Figure S6. Illustrative scheme of hyaluronic acid (HA) crosslinking with EDC and adipic acid dihydrazide (AAD).

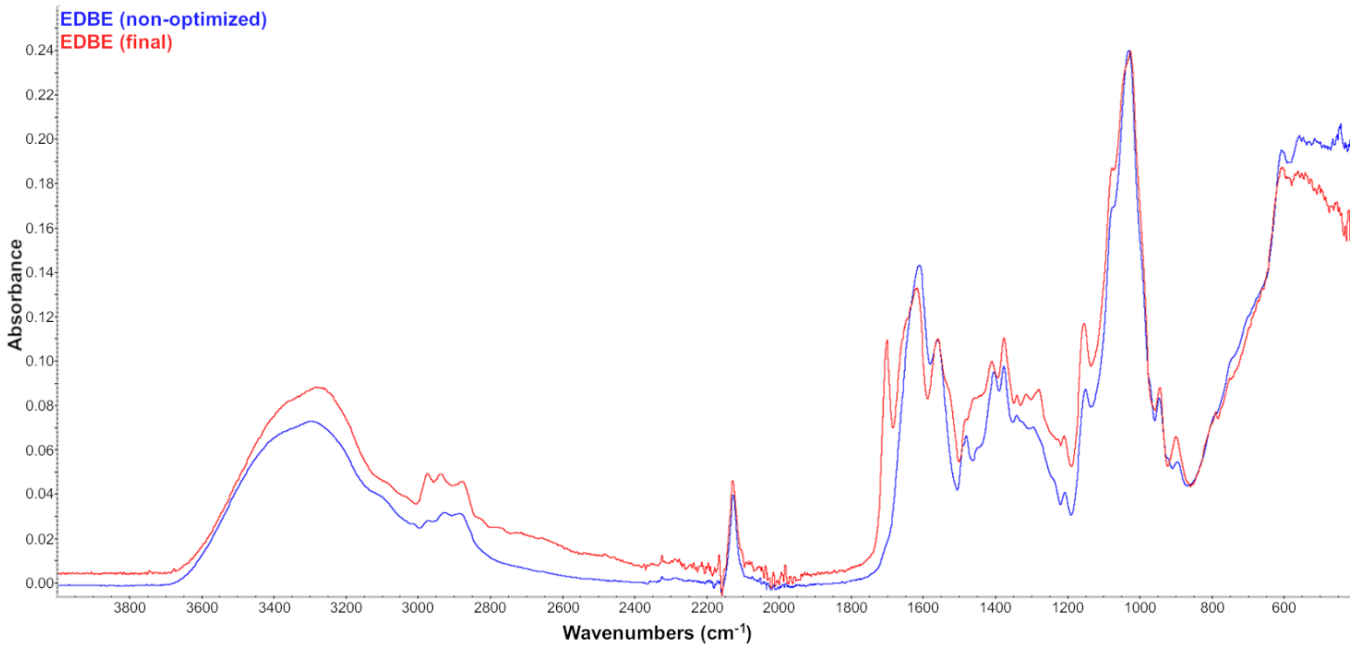

Figure S7. FT-IR spectra of the EDBE-HANPs prepared by Protocol no. 3. The blue spectrum represents the non-optimized nanoparticles, and the red spectrum represents the HANPs after the optimization.

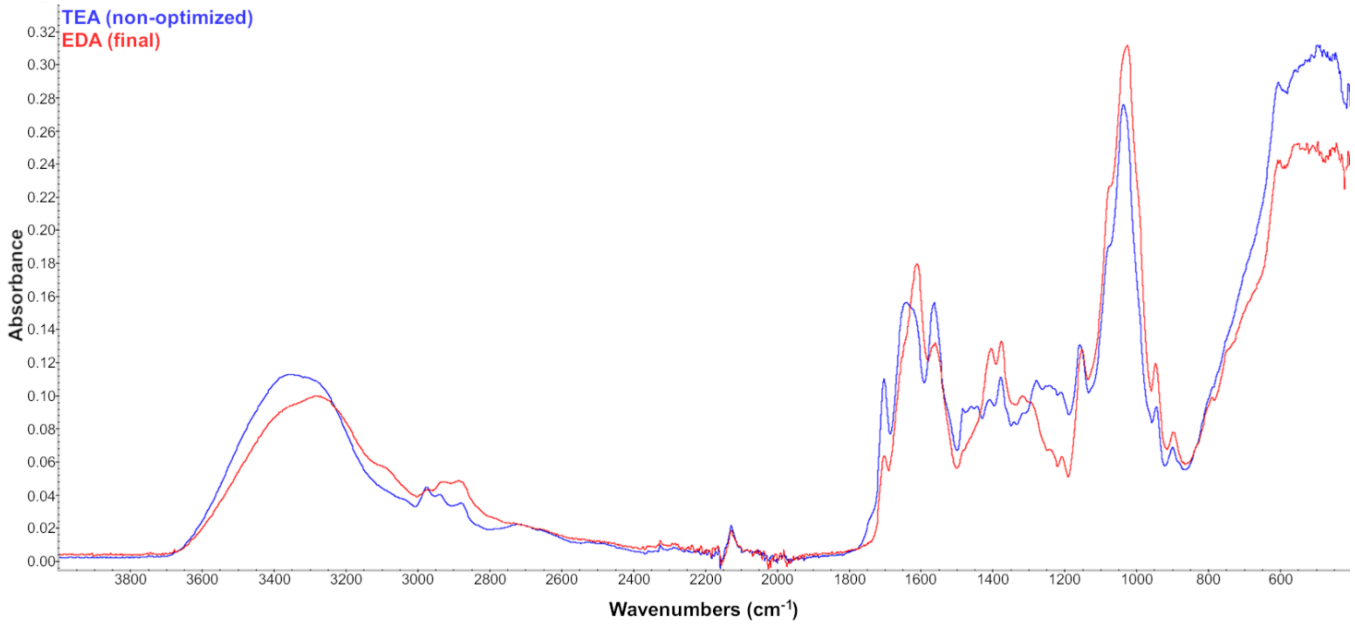

Figure S8. FT-IR spectra of the HANPs prepared with TEA and, consequently, EDA-HANPs prepared by Protocol no. 4. The blue spectrum represents the non-optimized nanoparticles, and the red spectrum represents the HANPs after optimizations.

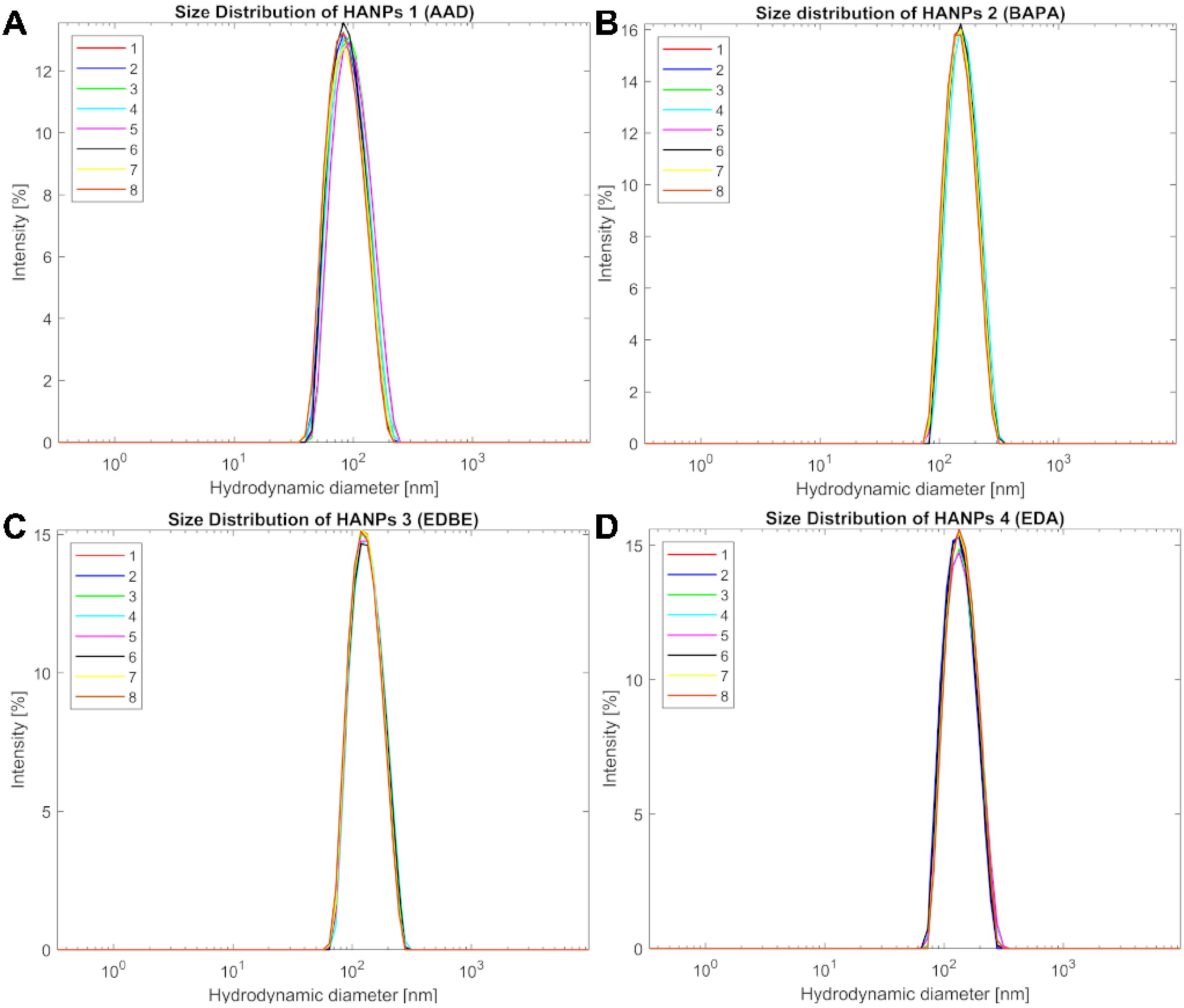

Figure S9. The size distribution measured by DLS ($n = 8$) of **(A)** Protocol no. 1 (AAD-HANPs), **(B)** Protocol no. 2 (BAPA-HANPs), **(C)** Protocol no. 3 (EDBE-HANPs), and **(D)** Protocol no. 4 (EDA-HANPs).

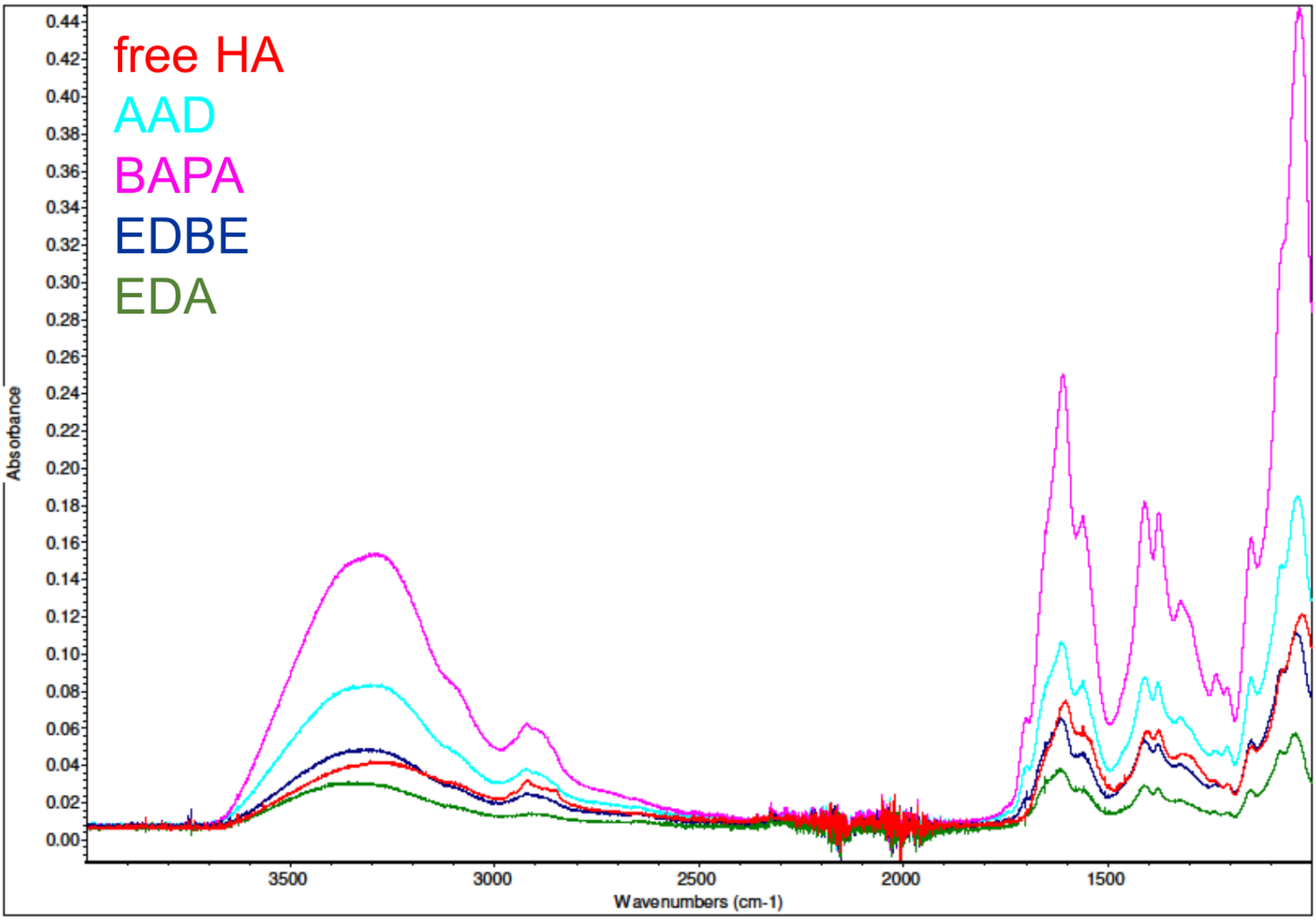

Figure S10. FT-IR spectra. Comparison of free hyaluronic acid (free HA) and AAD-, BAPA-, EDBE-, and EDA-HANPs.

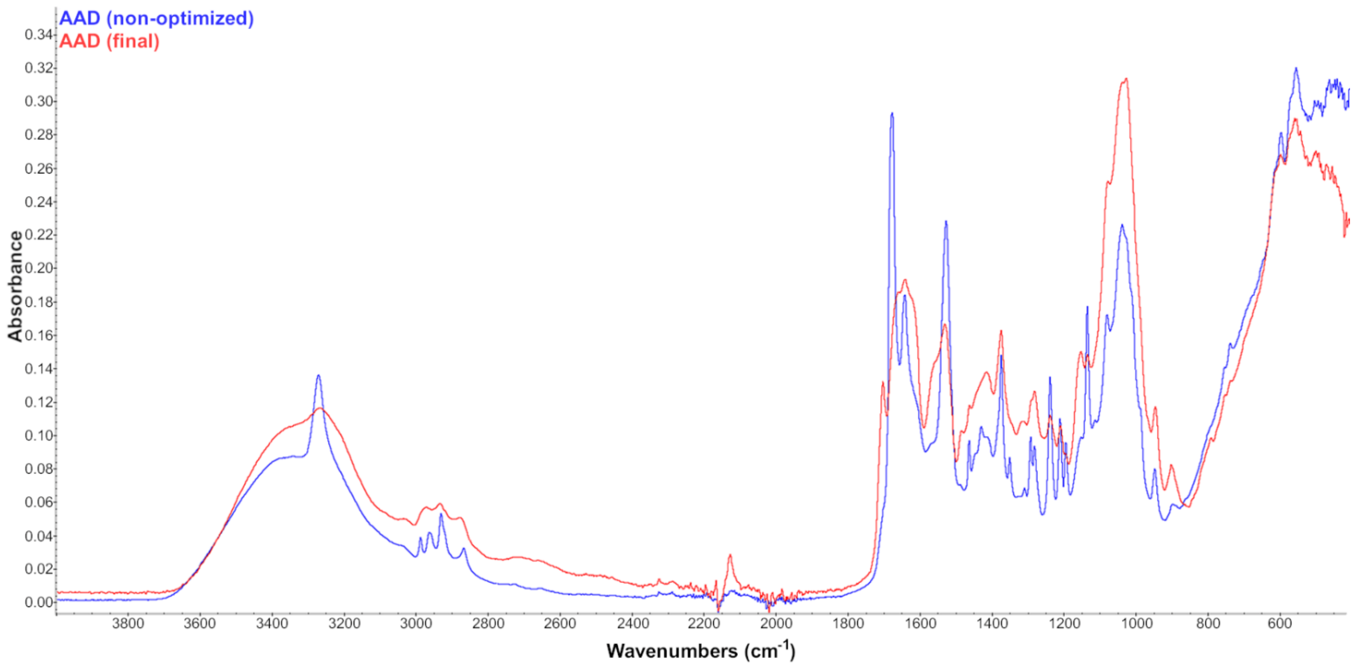

Figure S11. FT-IR spectra of the AAD-HANPs prepared by Protocol no. 1. The blue spectrum represents the non-optimized nanoparticles, and the red spectrum represents the HANPs prepared by the optimized protocol.

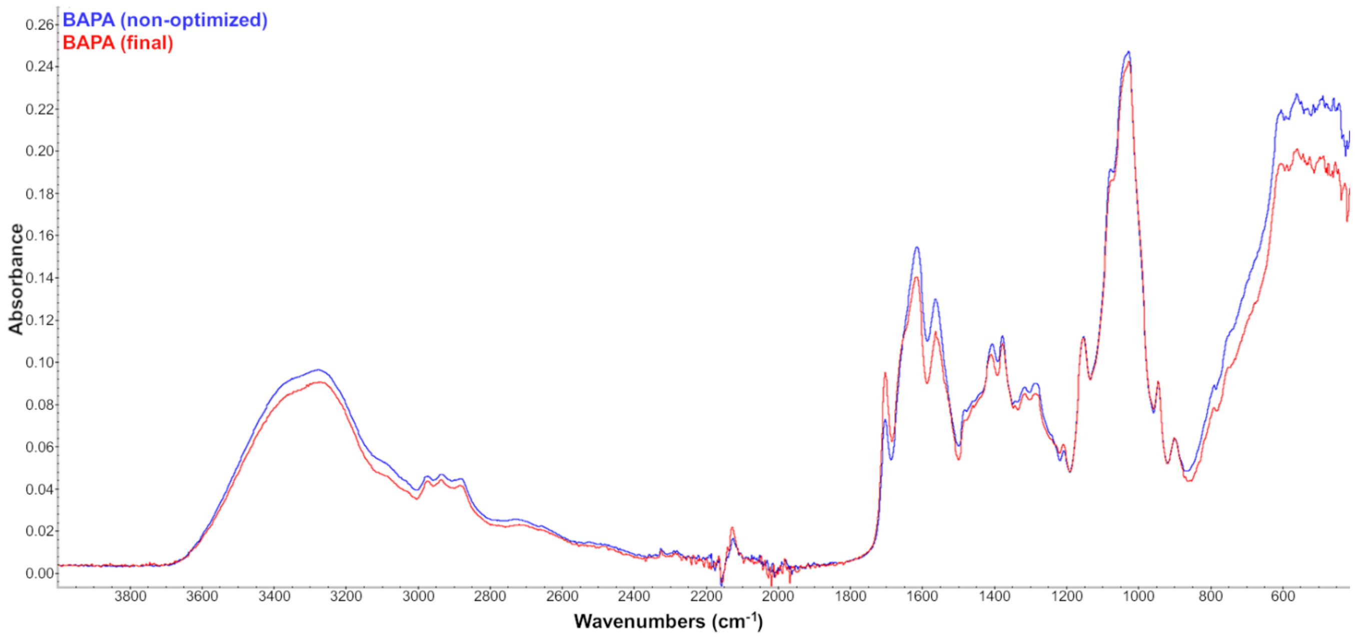

Figure S12. FT-IR spectra of the BAPA-HANPs prepared by Protocol no. 2. The blue spectrum represents the non-optimized nanoparticles, and the red spectrum represents HANPs prepared by optimized protocol.

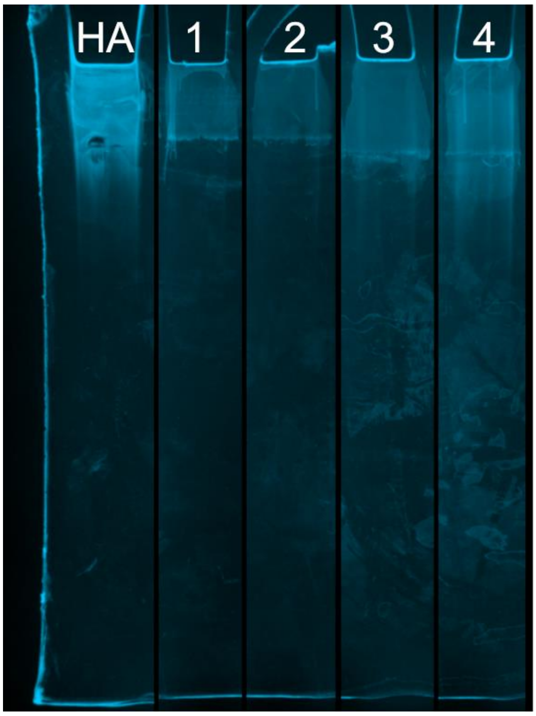

Figure S13. Image of a polyacrylamide gel from TBE-PAGE - 15% gel, stained with Alcian blue and silver staining. **HA** - free hyaluronic acid, **1** – AAD-HANPs, **2** – BAPA-HANPs, **3** – EDBE-HANPs, and **4** – EDA-HANPs.

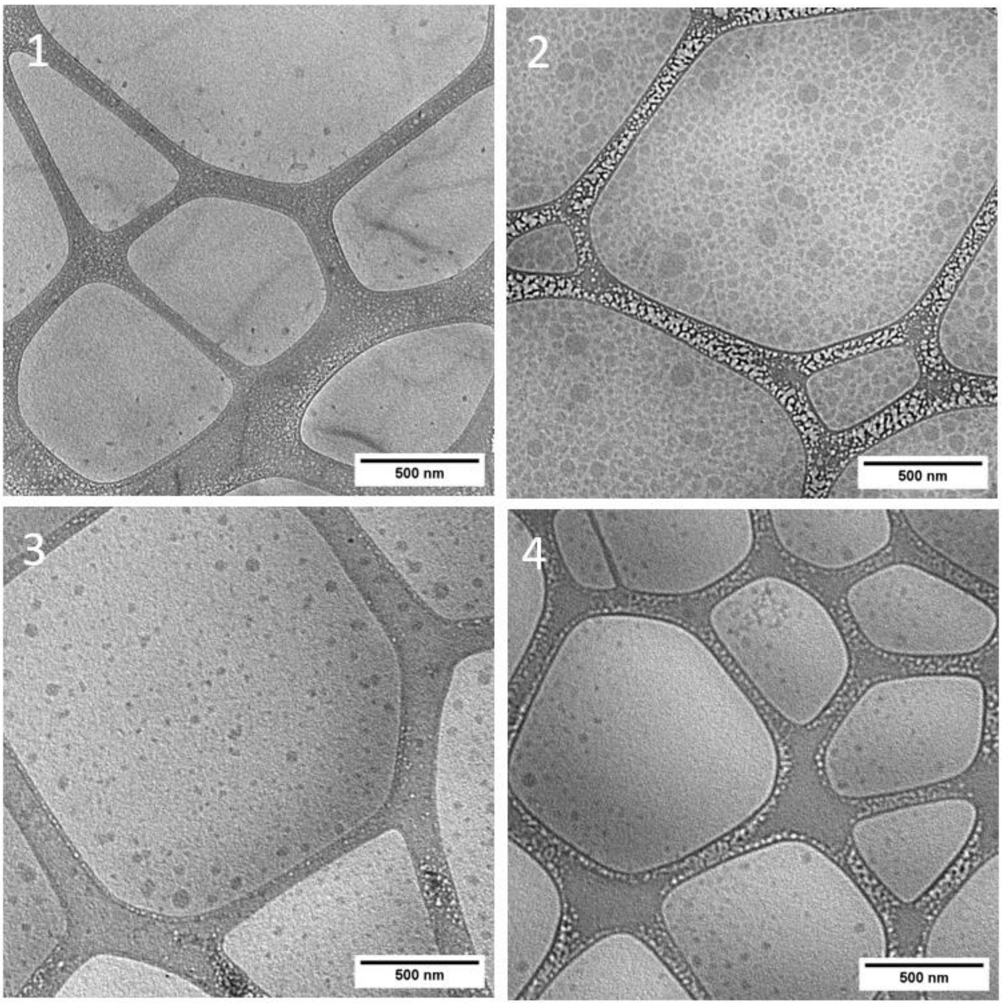

Figure S14. The images of HANPs obtained by cryo-TEM. **(1)** AAD-HANPs, **(2)** BAPA-HANPs, **(3)** EDBE-HANPs, and **(4)** EDA-HANPs.

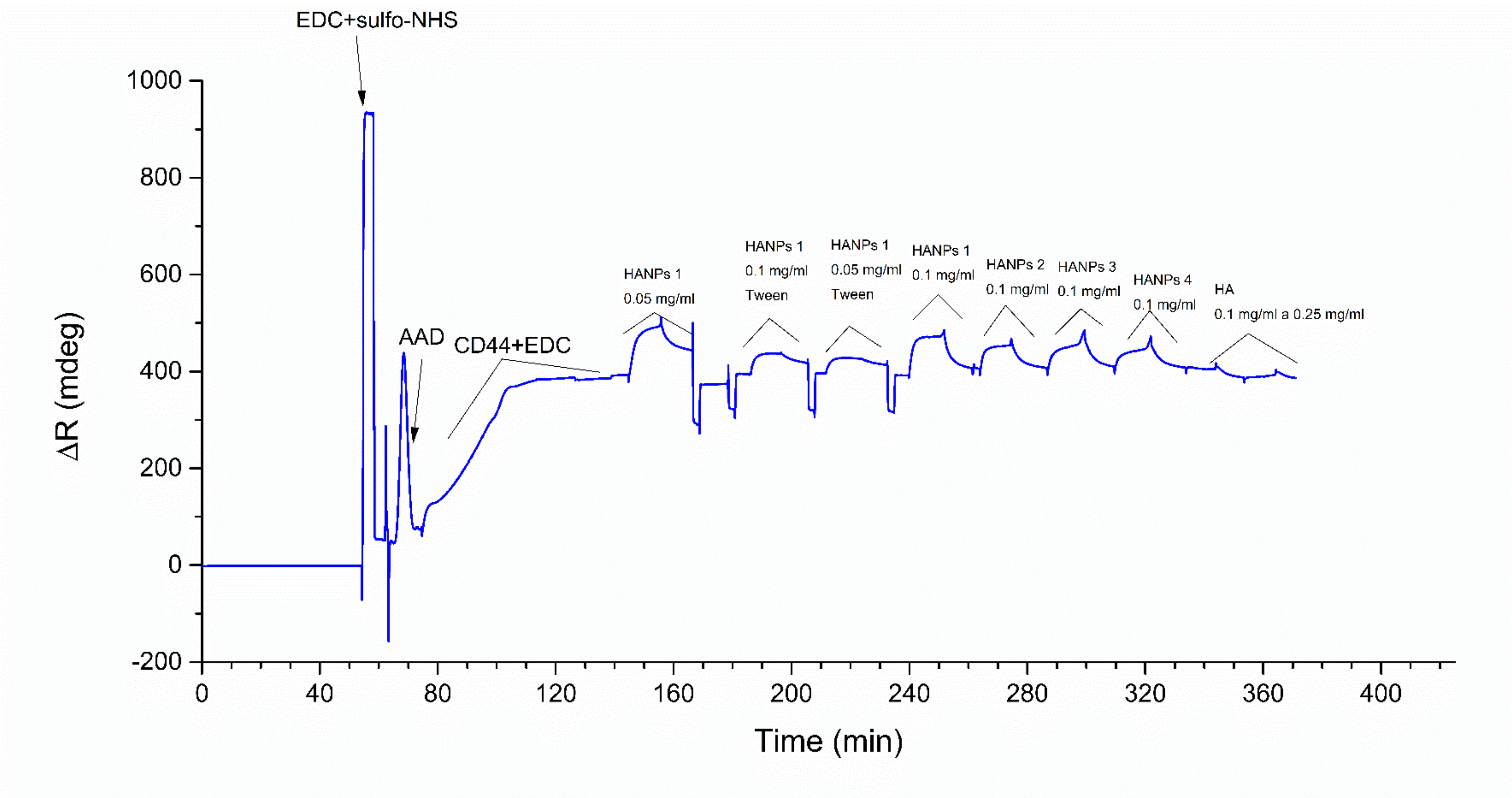

Figure S15. Sensorgram of the entire SPR measurement. Measurement affinity of HA and HANPs to CD44. The spectrum represents the difference between the signal on a specific channel (with CD44) and a reference channel (without CD44). HANPs 1 are AAD-HANPs, HANPs 2 are BAPA-HANPs, HANPs 3 are EDBE-HANPs, and HANPs 4 are EDA-HANPs.

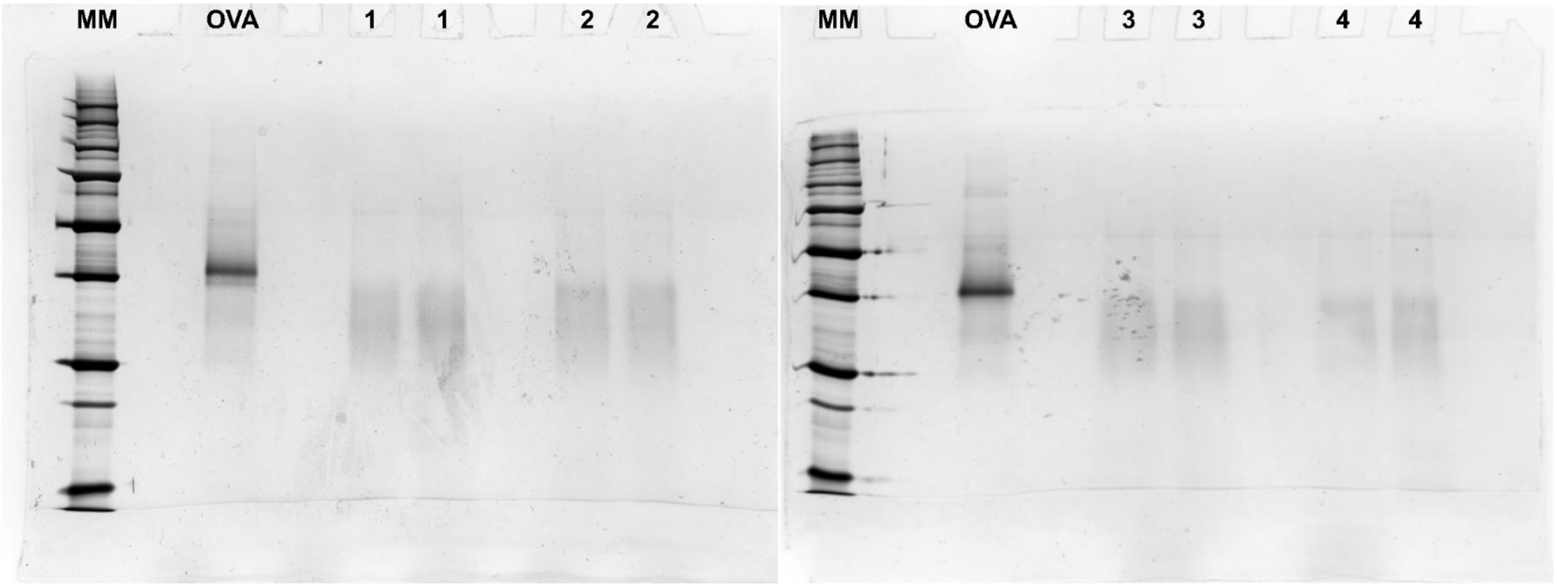

Figure S16. Images of gels from standard SDS-PAGE with silver staining to estimate the binding capacity of HANPs. **MM** – standard of molecular weights; **OVA** – original solution of ovalbumin used for binding; **1** – OVA-AAD-HANPs in doublet; **2** – OVA-BAPA-HANPs in doublet; **3** – OVA-EDBE-HANPs in doublet and **4** – OVA-EDA-HANPs in doublet. The binding of ovalbumin was performed by activation of HANPs (3 mg) by EDC (3 mg/200 µL ddH_2O). After 5 min incubation upon slow speed rotation, the sulfo-NHS solution (0.48 mg/200 µL ddH_2O) together with the ovalbumin solution (60 µg/1 mg HANPs) were added. The incubation upon slow speed rotation at 4°C for 16 hours followed.
